# SIGLEC-5/14 Inhibits CD11b/CD18 Integrin Activation and Neutrophil-Mediated Tumor Cell Cytotoxicity

**DOI:** 10.3390/ijms242417141

**Published:** 2023-12-05

**Authors:** Panagiota Bouti, Colin Blans, Bart J. A. M. Klein, Debarati Shome, Reza Nadafi, Michel Van Houdt, Karin Schornagel, Paul J. J. H. Verkuijlen, Virginie Roos, Rogier M. Reijmers, Robin Van Bruggen, Taco W. Kuijpers, Hanke L. Matlung

**Affiliations:** 1 Department of Molecular Hematology, Sanquin Research and Landsteiner Laboratory, Amsterdam UMC, University of Amsterdam, 1066 CX Amsterdam, The Netherlands; 2 LUMICKS, Paalbergweg 3, 1105 AG Amsterdam, The Netherlands; 3 Department of Pediatric Immunology and Infectious Diseases, Emma Children’s Hospital, Academic Medical Center, University of Amsterdam, 1105 AZ Amsterdam, The Netherlands

**Keywords:** neutrophil ADCC, SIGLEC, sialic acid, checkpoint blockade, antibody therapy

## Abstract

Since the successful introduction of checkpoint inhibitors targeting the adaptive immune system, monoclonal antibodies inhibiting CD47-SIRPα interaction have shown promise in enhancing anti-tumor treatment efficacy. Apart from SIRPα, neutrophils express a broad repertoire of inhibitory receptors, including several members of the sialic acid-binding receptor (SIGLEC) family. Here, we demonstrate that interaction between tumor cell-expressed sialic acids and SIGLEC-5/14 on neutrophils inhibits antibody-dependent cellular cytotoxicity (ADCC). We observed that conjugate formation and trogocytosis, both essential processes for neutrophil ADCC, were limited by the sialic acid-SIGLEC-5/14 interaction. During neutrophil-tumor cell conjugate formation, we found that inhibition of the interaction between tumor-expressed sialic acids and SIGLEC-5/14 on neutrophils increased the CD11b/CD18 high affinity conformation. By dynamic acoustic force measurement, the binding between tumor cells and neutrophils was assessed. The interaction between SIGLEC-5/14 and the sialic acids was shown to inhibit the CD11b/CD18-regulated binding between neutrophils and antibody-opsonized tumor cells. Moreover, the interaction between sialic acids and SIGLEC-5/14-consequently hindered trogocytosis and tumor cell killing. In summary, our results provide evidence that the sialic acid-SIGLEC-5/14 interaction is an additional target for innate checkpoint blockade in the tumor microenvironment.

## 1. Introduction

Current anti-cancer immunomodulatory approaches mainly engage cells of the adoptive immune system. Although T cell therapies have shown substantial clinical efficacy, the majority of cancer patients cannot benefit from these treatments [1,2]. Emerging evidence highlights the potential of innate immune system cells to interface with tumor cells, yielding both direct tumoricidal effects and indirect contributions to the priming and infiltration of CD8+ T cells [3]. Specifically, expression of Fc receptors (FcRs) on NK cells, macrophages and neutrophils induce antibody-mediated responses, such as antibody-dependent cellular phagocytosis (ADCP) or antibody-dependent cellular cytotoxicity (ADCC) [4]. In addition, the uptake of tumor-associated antigens induces antigen-cross presentation and tumor antigen release [5]. Therefore, a shift in paradigm towards therapies that exploit the innate immune system may enhance the anti-cancer response by establishing a multifaceted framework for effective tumor control.

Immune checkpoint inhibitor therapy (ICT) involves the disruption of interactions between tumor and immune cells, which prevent anti-tumor functions. Glycans are monosaccharide (sugar) chains that are attached at the terminal residues of proteins, lipids, or nucleic acids [6]. Alterations of glycans, including upregulation of cancer-associated sialylated glycans, are observed in several cancer types, and lead to increased metastasis and therapeutic resistance [7,8,9,10]. Binding of specific immune receptors, inhibitory sialic acid-binding receptors (SIGLECs), to these sialic acids promote immunosuppressive signaling, thereby providing increased opportunities for cancer cells to evade detection and removal by the immune system [11]. The SIGLEC family is comprised of 14 members, of which 9 contain an intracellular immune receptor tyrosine-based inhibition motif (ITIM) or ITIM-like motif, and 3 can induce activating signals due to interaction to DAP10/12, which carry an immune receptor tyrosine-based activation motif (ITAM) [12,13]. The binding of the ITIM-containing SIGLECs to sialic acids, initiates a downstream inhibitory signal via the recruitment of the SH2 domain-containing protein tyrosine phosphatases SHP-1 and SHP-2 [14,15]. As a result, sialic acid-SIGLEC interactions can interfere with cellular responses and may therefore also inhibit immune-mediated anti-tumor activity [16].

In line with this, in vitro and in vivo studies that investigated (engineered) hypersialylated cancer cells showed restricted NK and T cell killing of their target cells by engaging SIGLEC-7 and SIGLEC-9, respectively [17,18]. Furthermore, human polymorphisms that result in reduced SIGLEC-9 binding to sialic acids were correlated with improved survival for non-small cell lung cancer (NSCLC) patients [19]. Additionally, macrophage phagocytic activity of tumor cells was enhanced by inhibiting the CD24-SIGLEC-10 interaction, whereas inhibition of SIGLEC-7 expression by murine macrophages resulted in reduced neuroblastoma volume [20,21]. Results from these studies, amongst others, have raised possibilities for targeting sialylation to boost treatment response, and several compounds directed against the sialic acid-SIGLEC interactions are currently in clinical trials (NCT05259696, NCT03665285, NCT04699123) [22].

Current studies have also started focusing on targeting innate immune cells including neutrophils. Neutrophils are present in the tumor microenvironment [23], and besides their immunosuppressive functions, they are capable of killing antibody-opsonized tumor cells by ADCC instead of ADCP. This ADCC process relies on trogocytosis, initiated by the binding of a tumor-opsonizing antibody to the Fc receptors on the neutrophil and the active CD11b/CD18 integrins [24,25,26,27].

Neutrophils express SIGLEC-5, SIGLEC-9, and SIGLEC-14 which recognize sialylated glycans in an α2,3, α2,6, and α2,8 linkage conformation [28,29]. Whereas the inhibitory SIGLEC-5 and SIGLEC-9 proteins contain ITIM motives in their cytoplasmic tail, SIGLEC-14 associates with DAP12 in the plasma membrane to initiate an activating signal [30,31]. Even though SIGLEC-5 and SIGLEC-14 share over 99% homology at the first two Ig-like extracellular domains with identical glycan binding preferences, studies using SIGLEC-Fc-fusion proteins suggest that SIGLEC-14 binds to these sialic acids with higher avidity [31]. Recent studies reported the inhibitory role of SIGLEC-9 in neutrophil tumor killing capacity [19,32,33], although the underlying mechanism remained elusive.

In this study we focused on SIGLEC-5/14 and demonstrate that neutrophil ADCC is limited due to the sialic acid-SIGLEC-5/14 interaction. Inhibition of this interaction results in strengthening of the CD11b/CD18-mediated neutrophil-tumor cell conjugate formation and improves trogocytosis-mediated neutrophil killing of solid tumors.

## 2. Results

### 2.1. Immune Receptors SIGLEC-5 and SIGLEC-14, and Genes Involved in Sialic Acid Metabolism Are Present in Human Solid Tumors

**Figure 1:**
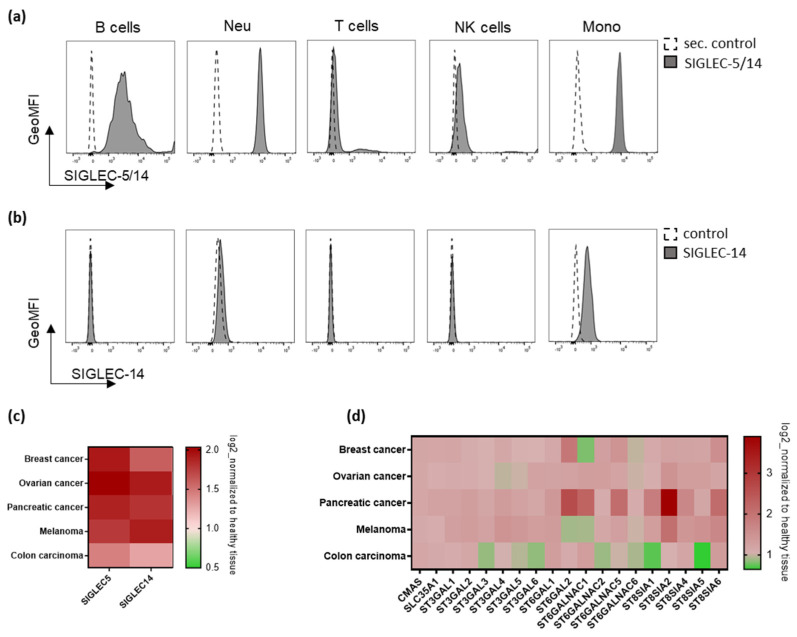
Immune receptors SIGLEC-5 and SIGLEC-14, and enzymes responsible for the biosynthesis of sialic acids are highly co-expressed in the tumor microenvironment. (**a**,**b**) Histograms from one representative example depict the expression of SIGLEC-5/14 (**a**) or SIGLEC-14 (**b**) in whole blood-derived CD19-positive B cells, CD16-positive neutrophils, CD3-positive T cells, CD56-positive NK cells, or CD14-positive monocytes. Histograms are shown as normalized to mode. (**c**) mRNA data expression of *SIGLEC-5* and *SIGLEC-14* genes in breast, ovarian, pancreatic, or melanoma cancers, normalized to healthy tissue. (**d**) mRNA data expression of genes regulating sialic acid metabolism in breast, ovarian, pancreatic, and melanoma cancers, normalized to healthy tissue. (**c**,**d**) GTEx vs. TCGA databases; normalized to GTEX).

To investigate the role of the interaction between sialic acids on tumor cells and SIGLEC-5/14 on neutrophils, we first verified the expression of SIGLEC-5 and SIGLEC-14 on human white blood cells using flow cytometry. We used two different antibodies, one detecting SIGLEC-5 and SIGLEC-14 (hereafter SIGLEC-5/14, cross reactivity due to high extracellular homology of SIGLEC-5 and SIGLEC-14), and one detecting SIGLEC-14 only. Specificity of each antibody was verified by overexpression of SIGLEC-5 and/or SIGLEC-14 on HEK293T cells (Supplemental Figure S1a,b). We observed SIGLEC-5/14 expression by B cells, neutrophils, NK cells, and monocytes and in lower levels by T cells. SIGLEC-14 expression was observed mainly by monocytes and to a minor extent by neutrophils (Figure 1a,b and Supplemental Figure S1c,d). Expression of SIGLEC-5/14 and SIGLEC-14 was further verified on isolated neutrophils (Supplemental Figure S1e,f). To evaluate the relevance of the sialic acid-SIGLEC-5/14 interaction in tumors, we made use of publicly available RNA sequencing data from the Genotype Tissue Expression project (GTEx) and The Cancer Genome Atlas (TCGA) databases. Heatmap analysis for the mRNA expression of SIGLEC-5 and SIGLEC-14 indicated the presence of these immune receptors in the tumor microenvironment (TME) of breast, ovarian, or pancreatic cancers, as well as melanomas and colon carcinomas, indicating the presence of macrophages as well as neutrophils (Figure 1c). In parallel, enzymes that are involved in sialic acid synthesis, activation, conjugation, or break down showed increased expression in all tumor-derived tissues tested, compared to healthy tissue (Figure 1d). These results illustrate the increased sialylation in the TME, accompanied by the presence of SIGLEC-5/14-expressing immune cells, including neutrophils.

### 2.2. Decreased Sialic Acid Expression on SKBR3 or A431 Solid Tumor Cells Enhances Neutrophil ADCC

**Figure 2:**
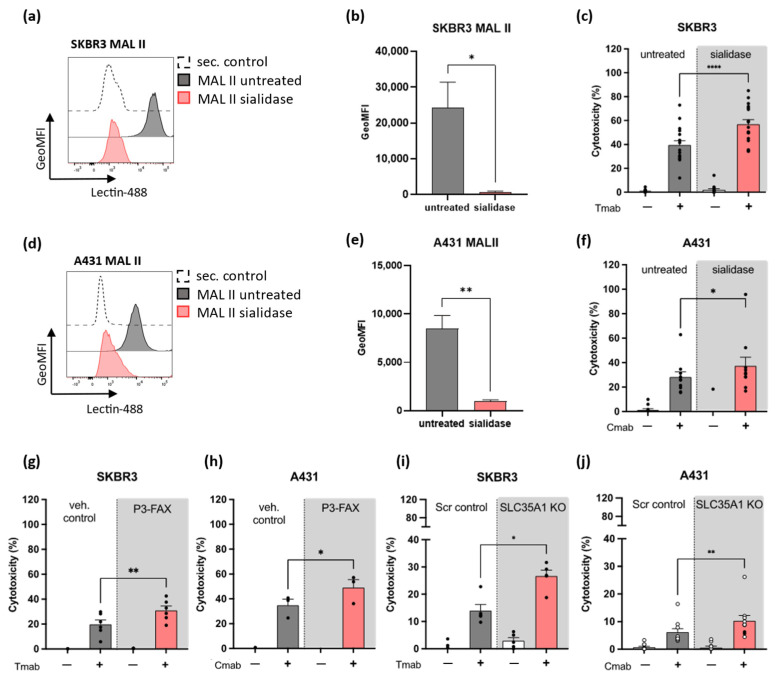
Inhibition of sialic acid expression on tumor cells enhances neutrophil ADCC. (**a**,**b**) Binding of Maackia amurensis lectin II (MAL II) to SKBR3 cells untreated or treated with sialidase (3 independent experiments). (**a**) Histogram from one representative example indicates the efficacy of sialidase treatment by MAL II binding. Histogram is shown as normalized to mode. (**c**) Neutrophil ADCC against SKBR3 cells in the presence or absence of opsonizing antibody trastuzumab and/or sialidase-treated tumor cells (12 donors from 6 independent experiments). (**d**,**e**) Binding of MAL II to A431 cells in the presence or absence of sialidase treatment (3 independent experiments). (**d**) Histogram from one representative experiment indicates MAL II binding in untreated or sialidase-treated A431 cells. Histogram is shown as normalized to mode. (**f**) Neutrophil ADCC against A431 cells in the presence or absence of opsonizing antibody cetuximab and/or sialidase-treated tumor cells (9 donors from 5 independent experiments). MAL II lectin binding was used in each experiment to verify the sialidase effect. (**g**,**h**) Neutrophil ADCC in the presence of tumor cells pre-treated with the sialyltransferase inhibitor P-3F_AX_-Neu5Ac (P3-FAX) or vehicle control (**g**: 6 donors from 2 independent experiments; **h**: 3 donors from 1 experiment). MAL II lectin binding was examined in each experiment to verify the P3-FAX effect. (**i**,**j**): ADCC was examined in the presence or absence of Sialic acid transporter Solute Carrier Family 35 Member A1 (SLC35A1), using SKBR3 or A431 SLC35A1 knock out (KO) cells) (**i**: 5 donors from 2 independent experiments; **j**: 10 donors from 5 independent experiments). Neutrophils were stimulated with G-CSF/IFNγ overnight (graph **j** includes neutrophils stimulated with G-MCSF, open circles). The bars show mean± SEM. Statistics: (**b**,**e**) unpaired *t*-test; (**c**,**f**–**j**) paired *t*-test; ns, nonsignificant; *, *p* < 0.05; **, *p* < 0.01; ****, *p* < 0.0001. ADCC, antibody-dependent cellular cytotoxicity; Tmab, trastuzumab; Cmab, cetuximab.

Next, we performed neutrophil ADCC assays and used three approaches to diminish the sialic acid expression on tumor cells. First, we removed the sialic acids from Her2/neu-expressing breast carcinoma SKBR3 cells and the epidermal growth factor receptor (EGFR)-expressing epidermoid carcinoma A431 cells using a *Vibrio-cholerae*- derived sialidase, a sialic acid-specific glycosidase. The efficacy of the sialidase treatment was evaluated prior to each neutrophil cytotoxicity assay by staining for maackia amurensis lectin (MAL II), which recognizes sialic acids with α2,3- linked configuration (Figure 2a,b,d,e). The binding of MAL II was significantly decreased in both SKBR3 (Figure 2a,b) and A431 (Figure 2d,e) tumor cell lines. To ensure that the sialic acid expression was not restored throughout the cytotoxicity assays, SKBR3 and A431 cells were incubated with sialidase for 30 min and binding of MAL II was assessed immediately after the incubation period and after 4 h, spanning the incubation time of the ADCC. As shown by MAL II binding, the effect of sialidase treatment was still present after 4 h (Supplemental Figure S2a,b). Neutrophil ADCC against antibody-opsonized SKBR3 (Figure 2c) or A431 cells (Figure 2f) was significantly increased after treatment of the tumor cells with sialidase. The effect was only observed in the presence of tumor antigen-targeting monoclonal antibody, i.e., trastuzumab (Tmab) or cetuximab (Cmab), highlighting the specificity of antibody-mediated killing by neutrophils, even after sialic acid removal from the tumor cell surface.

Secondly, we used the cell-permeable sialyltransferase inhibitor P-3Fax-Neu5Ac (P3-FAX) to reduce sialic acid expression on SKBR3 and A431 tumor cells [34]. P3-FAX acts as a sialic acid analog and thereby blocks sialoglycan synthesis. As a third approach, we generated a sialic acid transporter solute carrier family 35 member A1 knockout (SLC35A1 KO) line of the tumor cells. SLC35A1 encodes the CMP-sialic acid transporter located in the Golgi apparatus, therefore this knockout approach abolishes the protein responsible for sialic acid transportation. The use of P3-FAX resulted in the depletion of α2,3-linked sialylation on the cell surface (Supplemental Figure S2c,e), leaving the antibody opsonization of the tumor antigens Her2/neu and EGFR unaltered (Supplemental Figure S2d,f). Similar results were obtained using the SLC35A1 KO cells (Supplemental Figure S2g–j). Neutrophil ADCC was evaluated after pre-incubation of tumor cells with P3-FAX (efficacy of the treatment was verified prior to each ADCC, as depicted in Supplemental Figure S2c,e), or alternatively after use of the SLC35A1 KO cells. Similarly to sialidase treatment, the cytotoxicity against antibody-opsonized SKBR3 and A431 cells was significantly increased after P3-FAX pre-incubation of the tumor cells and by using the SLC35A1 KO cells (Figure 2g–j), suggesting that tumor cell sialylation limits the efficacy of neutrophil ADCC.

### 2.3. Inhibition of Sialic Acid Interaction with SIGLEC-5/14 Enhances Neutrophil ADCC of Solid Tumor Cells

**Figure 3:**
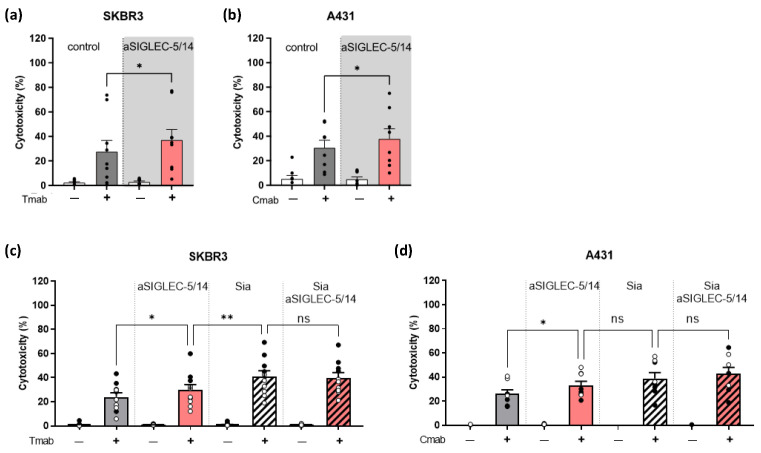
Blocking SIGLEC-5/14 increases neutrophil ADCC against solid tumor cells. (**a**–**d**) Neutrophil ADCC against SKBR3 (**a**,**c**) or A431 (**b**,**d**) cells, in the presence or absence of monoclonal antibody trastuzumab or cetuximab, respectively. Neutrophils were stimulated with G-CSF/IFNγ overnight and pre-incubated with human SIGLEC-5/14 blocking antibody, where indicated (**a**: 15 donors from 8 independent experiments; **b**: 13 donors from 7 independent experiments). (**c**,**d**) Tumor cells were pre-treated with sialidase (closed circles), or P3-FAX (open circles) (together referred to as Sia) (**c**: 10 donors from 4 independent experiments; **d**: 8 donors from 3 independent experiments). Neutrophils were stimulated with G-CSF/IFNγ overnight. The bars show mean ± SEM. Statistics: (**a**,**b**) paired *t*-test; (**c**,**d**) one-way ANOVA with Sidak correction; ns, not significant; *, *p* < 0.05; **, *p* < 0.01. ADCC, antibody-dependent cellular cytotoxicity; Tmab, trastuzumab; Cmab, cetuximab.

To investigate the contribution of SIGLEC-5 and SIGLEC-14 in restricting neutrophil ADCC, we used a neutralizing antibody against SIGLEC-5/14. Inhibition of SIGLEC-5/14 increased the ADCC towards antibody-opsonized tumor cells (Figure 3a,b). These results replicated the improved neutrophil-mediated tumor cell killing that was observed when the sialic acid expression on tumor cells was reduced. To explore the involvement of other SIGLECs in neutrophil ADCC, we combined treatment with sialidase or P3-FAX (collectively referred to as Sia) and pre-incubation of neutrophils with SIGLEC-5/14 blocking antibody. By using the Her2/neu-positive SKBR3 cells, we observed that the cytotoxic effect after reduction of the tumor cell-sialic acids was significantly higher, in comparison to treatment with the SIGLEC-5/14 alone (Figure 3c). Combined removal of the sialic acids on the tumor cells and the inhibition by the SIGLEC-5/14 antibody did not further enhance the killing efficacy. Neutrophil ADCC using the EGFR-positive A431 cells showed a similar trend, although less significant (Figure 3d).

### 2.4. Sialic Acid-SIGLEC-5/14 Interaction Regulates Neutrophil Conjugate Formation and Trogocytosis by Preventing the High Affinity Conformation of CD11b/CD18

**Figure 4:**
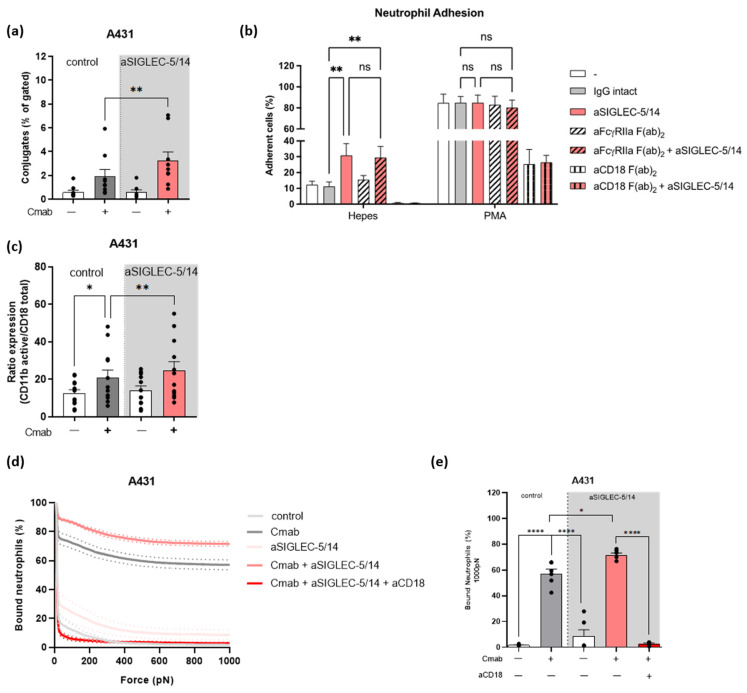
Inhibition of the interaction between sialic acids and SIGLEC-5/14 increases the activation of CD11b/CD18 in the context of neutrophil ADCC. (**a**) Conjugate formation measured as the percentage of gated population (neutrophils/calcein violet AM- and tumor cells/cell tracker red- positive cells) between neutrophils and A431 tumor cells. Neutrophils were pre-incubated with SIGLEC-5/14 blocking antibody and tumor cells were opsonized with cetuximab, where indicated (9 donors from 5 independent experiments). (**b**) Adhesion of unstimulated or Phorbol 12-myristate 13-acetate (PMA)-stimulated neutrophils. Neutrophils were pre-incubated with intact IgG antibody as an isotype control, or SIGLEC-5/14, FcγRIIa F(ab)_2_, FcγRIIa F(ab)_2_ and SIGLEC-5/14, CD18 F(ab)_2_ and SIGLEC 5-14 or CD18 F(ab)_2_ blocking antibodies. (**c**) Flow cytometry analysis displaying the ratio of activated epitope of CD11b (CBRM1/5) towards the total CD18 expression. Neutrophils were pre-incubated with SIGLEC-5/14 blocking antibody, and tumor cells were opsonized with cetuximab, where indicated (12 donors from 6 independent experiments). (**d**) Avidity curve shows the percentage of neutrophils bound to A431 in the presence or absence of cetuximab after application of increasing acoustic force (pN). (**e**) Bar graph presentation of the neutrophils bound to A431 after force application of 1000 pN. Statistics: (**a**) paired *t*-test; (**b**,**c**) one-way ANOVA with Tukey correction; (**e**) one-way ANOVA with Sidak correction; ns, nonsignificant; *, *p* < 0.05; **, *p* < 0.01, **** *p* < 0.0001. Cmab, cetuximab.

Neutrophil ADCC of tumor cells requires the tight interaction between neutrophil and tumor cell, followed by trogocytosis of the tumor cell membrane [25]. First, neutrophil and A431 tumor cell conjugate formation was evaluated in the presence or absence of the opsonizing antibody cetuximab and SIGLEC-5/14 blocking antibody, using imaging flow cytometry. Conjugate formation reflected the ADCC response as it was increased in the presence of the SIGLEC-5/14 blocking antibody (Figure 4a and Supplemental Figure S3a). In addition, we observed a significant enhancement in trogocytosis, measured by flow cytometry as membrane transfer from antibody-opsonized tumor cells to neutrophils, when the SIGLEC-5/14 blocking antibody was present (Supplemental Figure S3b–e).

Both the conjugate formation as well as trogocytosis are processes mediated by activation of CD11b/CD18 [25,27,35]. Therefore, we next assessed the CD18-dependent neutrophil adhesion using the SIGLEC-5/14 blocking antibody. At steady state, neutrophils adhered only weakly. In contrast, addition of the SIGLEC-5/14 antibody significantly increased neutrophil adhesion, which was dependent on CD18 (Figure 4b). To rule out the potential binding of the SIGLEC-5/14 antibody (IgG1) to the FcγIIa receptor (FcγRIIa) on neutrophils (known as the Kurlander phenomenon) [36], we pre-incubated neutrophils with F(ab’)_2_ antibodies against FcγRIIa. The presence of SIGLEC-5/14 rather than FcγRIIa blocking antibody increased neutrophil adhesion (Figure 4b). Together, these results show that antibody-mediated inhibition of SIGLEC-5/14 increases the adhesion of neutrophils, which is mediated through CD11b/CD18.

Next, we explored the levels of CD11b/CD18 high affinity conformation by using an antibody that detects the activation epitope (clone CBRM1/5) of CD11b. After co-incubation of neutrophils with A431 cells, we observed an increase in the ratio of active versus total integrin levels in the presence of opsonizing antibody cetuximab, indicating an increased affinity conformation of CD11b/CD18 (Figure 4c). This ratio was further increased in the presence of SIGLEC-5/14 antibody. (Figure 4c). Cetuximab alone did not induce increased expression of the activation epitope in CD11b/CD18 (Supplemental Figure S3f). In addition, in the tested conditions the total levels of CD18 remained unaltered (Supplemental Figure S3g). These findings strongly suggest that the full activation of CD11b/CD18 is restricted by the sialic acid-SIGLEC-5/14 interaction during neutrophil-tumor cell interaction.

### 2.5. Sialic Acid-SIGLEC-5/14 Interactions Restrict Neutrophil-Tumor Cell Interactions

To assess the impact of sialic acid-SIGLEC-5/14 interactions on neutrophil-tumor cell conjugate formation, we used the Z-Movi cell avidity assay [37], which acts as a predictor of effector cell cytotoxic efficacy against tumors, and measured the forces required to detach the neutrophils from A431 cells (Figure 4d,e). Expression of CD11b, CD18, and SIGLEC-5/14 remained similar among donors (Supplemental Figure S3h) and conjugate formation of the tested donors followed a similar trend to the one observed previously (Supplemental Figure S3i and Figure 4a). In the absence of cetuximab, application of less than 200 pN force was enough to pull away all the neutrophils from the A431 cells. Addition of cetuximab to the tumor cells significantly enhanced the interaction between the two cell types, as up to 60% of neutrophils remained bound to the tumor cells after application of force up to 1000 pN (Figure 4d,e). The interaction between neutrophils and antibody-opsonized tumor cells was further strengthened after pre-incubation of neutrophils with the SIGLEC-5/14 blocking antibody, while this was completely abrogated by the addition of a blocking antibody against CD18 (Figure 4d,e). These results demonstrate that inhibition of the sialic acid-SIGLEC-5/14 interaction leads to enhanced neutrophil avidity due to increased CD11b/CD18 activation.

## 3. Discussion

Hypersialylation has been demonstrated to play a role in modulating immune cell responses to tumor cells [38]. SIGLECs, expressed by immune cells including neutrophils, act as sialic acid binding receptors and as such have the potential to suppress anti-tumor responses. This study demonstrates that by inhibiting the sialic acid-SIGLEC-5/14 interaction, the effectiveness of CD11b/CD18-mediated neutrophil cytotoxic mechanism against tumor cells can be enhanced. This enhancement is achieved through a strengthened binding of neutrophils to the tumors, leading to a more effective trogocytosis, and eventually cytotoxicity of antibody-opsonized tumor cells.

By using sialidase treatment, the sialyltransferase inhibitor P3-FAX, or SLC35A1 KO tumor cells to remove or prevent sialic acid expression on the surface of tumor cells, we demonstrate a notable enhancement in neutrophil-mediated killing efficacy. These findings suggest that inhibition of sialylation can improve neutrophil ADCC in tumor-opsonized conditions. Inhibition by SIGLEC-5/14 blocking antibodies mimicked the aforementioned response. However, combined sialic acid and SIGLEC-5/14 inhibition (referred to as Sia) further enhanced neutrophil ADCC compared to SIGLEC-5/14 blocking antibody alone, suggesting that SIGLEC-9 may also inhibit neutrophil cytotoxicity, as was most recently reported with IgA- or IgG-opsonized solid tumor cells [32,39]. Although removal of sialic acids can reduce the negative electrostatic barrier of tumor cells and affect their interaction with effector cells, as it has been shown for human macrophages [40], antibody opsonization was strictly required for initiating effective killing of tumor cells by neutrophils.

Our results show that SIGLEC-5/14 restricts CD11b/CD18 activation in the context of neutrophil and tumor cell interaction. In a similar fashion, the well-established innate checkpoint inhibitor SIRPa has been shown to restrict neutrophil CD11b/CD18 activation, and accompanying ADCC [25,27,41]. In macrophages, integrin activation was shown to be inhibited by positioning of SIRPa in the phagocytic synapse after ligation of tumor-expressed CD47. Based on these findings and our own data, we could speculate that the regulation of CD11b/CD18 activation by SIGLEC-5/14 operates in a similar fashion [42]. Nevertheless, we cannot rule out the possibility of SIGLEC-5/14-mediated restriction of integrin activation in cis [43].

Our findings indicate that SIGLEC-5 and/or -14 are responsible for dampening the neutrophil anti-tumor killing response and this could be extended to other innate immune cells, as also macrophages and NK cells express SIGLEC-5/14. It has been shown that inhibition of the sialic acid-SIGLEC interaction enhances NK cell-mediated killing against trastuzumab-opsonized SKBR3 [44]. Precise targeting of sialic acids by the use of antibody-recombinant sialidase conjugates has also shown promising results [44,45]. Overall, these observations suggest that the cytotoxic potential of several cells of the innate immunity could benefit from inhibition of the sialic acid-SIGLEC-5/14 interaction, improving their anti-tumor properties and, as studies also suggest, contributing to a more efficient adaptive cell response against tumor cells by antigen release or cross-presentation [46,47,48].

A limitation of our study is that we were unable to distinguish between the relative contributions of SIGLEC-5 and SIGLEC-14. These receptors share over 99% homology in their first two Ig-like extracellular domains, including their sialic acid-ligand binding preference [31]. The intracellular regions differ between the two receptors. Two ITIM motives are present in SIGLEC-5, while SIGLEC-14 associates with DAP12 for cellular activation [31]. Since our study identifies inhibitory effects on ADCC of intact SIGLEC-5/14-sialic acid binding, it seems most likely that SIGLEC-5 signaling predominates in the neutrophil cytotoxic effector functions, at least under the conditions used [49]. Recently, SIGLEC-5, and to a lesser extent SIGLEC-14 mRNA levels, were found to be enhanced in polymorphonuclear myeloid-derived suppressor cells (PMN-MDSCs) isolated from peripheral blood of glioma patients, compared to healthy individuals [50]. In the same study, SIGLEC-5 expression was highly increased in glioma-infiltrating PMN-MDSCs but not in monocytic myeloid-derived suppressor cells (M-MDSCs), compared to the healthy control group. These results implicate that increased SIGLEC-5 engagement on neutrophils in the TME may shift neutrophil plasticity towards an MDSC phenotype. To exclude any potential role for SIGLEC-14, next to SIGLEC-5, better tools are needed to separate the two SIGLECs from each other during neutrophil ADCC.

In summary, our findings indicate that SIGLEC-5/14 plays a role in limiting neutrophil ADCC against solid tumors. This is achieved by inhibiting the high affinity conformation of CD11b/CD18, which consequently hinders the formation of a strong interaction between tumor cells and neutrophils, as demonstrated through dynamic acoustic force measurements. Lastly, selectively inhibiting the sialic acid-SIGLEC interaction in a tumor-specific manner, such as through the use of highly specific antibodies that target tumor antigens and sialylation on tumor cells [51,52], may enhance current antibody-based anti-tumor approaches and promote the involvement of innate cells, including neutrophils.

## 4. Materials and Methods

### 4.1. Neutrophil Isolation

Neutrophils were isolated from healthy donors using isotonic percoll density gradient centrifugation, as previously described [53]. Heparinized blood was 1:1 diluted in phosphate-buffered saline (PBS) + 10% trisodium citrate (TNC) and loaded on the isotonic percoll (1.076 g/mL, GE Healthcare Life Science, Chicago, IL, USA). After centrifugation (20 min, 938× *g*, room temperature), the pellet fraction was lysed (erythrocyte lysis with ice cold hypotonic ammonium chloride solution (155 mM NH_4_Cl (Merck, Burlington, MA, USA), 10 mM KHCO_3_ (Merck), 0.1 mM EDTA (Merck), in H_2_0 (Gibco, Grand Island, NY, USA))). Following thorough PBS washing, neutrophils were reconstituted in 5 × 10^6^ /mL in HEPES medium (20 mM 4-(2-hydroxyethyl)-1-piperazineethanesulfonic acid (HEPES; Sigma Aldrich, St. Louis, MO, USA), 132 mM NaCl (Fagron, Rotterdam, The Netherlands), 6 mM KCl (Merck), 1 mM MgSO_4_ (Merck), 1.2 mM K_2_HPO_4_ (Merck), 7 H20 (Gibco), pH 7.4 with 10 mol/L NaOH), supplemented with 5 g/L human albumin (Albuman, Sanquin Plasma Products, Amsterdam, The Netherlands), 5.5 mM glucose (Merck) and 1 mM CaCl_2_ (Calbiotech, El Cajon, CA, USA) (referred to as HEPES+ medium) [53]. Where indicated, neutrophil activation was achieved by stimulation with either 50 ng/mL interferon gamma (IFNγ, Peprotech, Cranbury, NJ, USA) and 10 ng/mL granulocyte-colony stimulating factor (G-CSF, Neupogen, Amgen, Thousand Oaks, CA, USA) for 1 h, 4 h, or overnight, or 10 ng/mL granulocyte-macrophage-colony stimulating factor (GM-CSF, Peprotech) for 30 min. In case of overnight incubation and prior to every experiment, the percentage of apoptotic cells was corrected by Annexin V staining measured by flow cytometry (BD Biosciences, Franklin Lakes, NJ, USA).

### 4.2. Cell Culture and Modifications

Human epidermal growth factor receptor (EGFR)-expressing epidermoid carcinoma cell line A431 (ATCC) was cultured using RPMI (Gibco) supplemented with 10% (*v*/*v*) fetal calf serum (Bodinco B.V., Alkmaar, The Netherlands), 2 mM L-glutamine (Sigma Aldrich), 100 U/mL penicillin (Sigma Aldrich), and 100 μg/mL streptomycin (Sigma Aldrich) (referred to as RPMI culture medium). HER2/neu-expressing breast cancer cell line SKBR3 (ATCC, Manassas, VA, USA) was cultured with Iscove modified Dulbecco media (IMDM, Thermo Fisher Scientific, Waltham, MA, USA), supplemented with 20% (*v*/*v*) fetal calf serum, 2 mM L-glutamine, 100 U/mL penicillin, and 100 μg/mL streptomycin (referred to as IMDM culture medium). Tumor antigen expression was routinely tested by flow cytometry. HEK293T cell line (ATCC) was cultured using Dulbecco’s Modified Eagle Medium (DMEM), supplemented with 10% (*v*/*v*) fetal calf serum, 2 mM L-glutamine, 100 U/mL penicillin, and 100 μg/mL streptomycin (referred to as DMEM culture medium). The cell lines were harvested using trypsin (Sigma Aldrich), maintained at 37 °C and 5% CO_2_ for up to 3 months, and tested negative for *Mycoplasma* using PCR.

A431 or SKBR3 SLC35A1 knock out (SLC35A1KO) cell lines were generated by lentiviral transduction with pLentiCrispR-v2, in which a guide RNA (gRNA) against *SLC35A1* or a scrambled (Scr) gRNA (5′ gcactaccagagctaactca 3′) was cloned. Transduced cells were selected with 1 μg/mL puromycin (Invivogen, San Diego, CA, USA) and knockout efficiency was determined by flow cytometry. A successful knockout was found using gRNA 1 (5′ ttctgtgatacacacggctg 3′) for A431 and gRNA 2 (5′ tgaacagcatacactaacga 3′) for SKBR3. Cell lines were routinely tested for maackia amurensis lectin II (MAL II- biotinylated, Vector Laboratories, Newark, CA, USA) binding using flow cytometry.

For optimization of the specificity of SIGLEC-5/14 and SIGLEC-14 antibodies we used the HEK293T cell line (ATCC) and overexpressed SIGLEC-5, SIGLEC-14 or both. The plasmid vectors pRRL PPT SFFV prester SIN (pSIN) contained *SIGLEC-5-HA* IRES *GFP* or *SIGLEC-14-V5*- IVS-IRES *Cherry* (Thermo Fisher Scientific, both sequences were codon optimized for expression in human cells) (IVS: intervening sequence from pIRESpuro2, used for enhanced stability of the mRNA), first cloned into pENTR1A, and then recombined using LR Clonase II (Thermo Fischer Scientific, Waltham, MA, USA) with pRRL PPT SFFV prester SIN (pSin) in which a Gateway Cassette was cloned previously. One μg of plasmid (33 μg/mL) was transiently transfected using 3 μg of PEI-Max 40K (0.1 mg/mL; Polysciences, Inc., Warrington, PA, USA). For co-transfections, 1 μg of each plasmid was used with 6 μg of PEI Max 40K. DMEM culture medium was refreshed the day after transfection, and SIGLEC expression levels were examined the day after, using flow Cytometry (LSRII, BD Biosciences). Successful transient expression of SIGLEC-5 and/or SIGLEC-14 was assessed using the green fluorescent protein (GFP) and the monomeric red fluorescent protein Cherry, respectively. Cells were first gated for GFP, Cherry, or GFP and Cherry expression, and SIGLEC expression was evaluated further.

### 4.3. Reagents and Antibodies

For image stream, trogocytosis, ADCC, and avidity assays, SKBR3 or A431 cells were opsonized with trastuzumab (IgG anti-HER2/neu, 5 μg/mL, Roche, Basel, Switzerland) or cetuximab (IgG anti-EGFR, 5 μg/mL, Merck KGaA, Schiphol-Rijk, The Netherlands), respectively. As indicated in the specific graphs, tumor cells were pre-treated with sialidase (Neuraminidase from Vibrio cholerae, Type II, enzyme catalytic activity was evaluated and used at 1 nmol/min, Sigma Aldrich) for 30 min at 37 °C and 5% CO_2_. Alternatively, A431 and SKBR3 cells were pre-treated with the sialyltransferase inhibitor *p*-3F_AX_-Neu5Ac (P3-FAX, EMD Millipore, St. Louis, MO, USA) in a concentration of 30 μg/mL for 72 h at 37 °C and 5% CO_2_ prior use. Dimethyl sulfoxide (DMSO) was used as vehicle control. Cells were washed and the efficacy of sialidase or P3-FAX treatment was evaluated with MAL II staining using flow cytometry. In the conditions indicated, neutrophils were pre-incubated with antibodies against human SIGLEC-5/14 (10 μg/mL, R&D systems), FcγRIIa F(ab’)_2_ (10 μg/mL, Ancell Corporation), CD18 F(ab’)_2_ or intact IB4 clone (10 μg/mL, Ancell Corporation), or purified IgG control (10 μg/mL, Life Technologies, Merelbeke, Belgium).

### 4.4. Flow Cytometry

SIGLEC expression on neutrophils was assessed using flow cytometry. Antibodies against human SIGLEC-5/SIGLEC-14 (SIGLEC-5/14, 10 μg/mL, R&D systems, McKinley Place NE, MN, USA), or SIGLEC-14 (20 μg/mL, R&D systems) were used, and antibody Alexa Fluor 647 F(ab’)_2_ goat anti-mouse (10 μg/mL, Invitrogen, Carlsbad, CA, USA) was used for secondary incubation when needed. Neutrophils were stained for negative IgG control (Diaclone, Besançon, France), Annexin V (BD Biosciences), FcγRIIIb (BD Biosciences), FcγRIIa or FcγRI (BioRad, Hercules, CA, USA), CD11b (BioRad) and CD18 (clone MEM48; Diaclone). For the detection of B cells, neutrophils, T cells, NK cells, or monocytes in whole blood after erythrocyte lysis the following antibodies were used: human CD19, CD16, CD3, CD56, or CD14 (BD Biosciences). Expression of maackia amurensis lectin II (MAL II- biotinylated, 5 μg/mL, Vector Laboratories) was evaluated on tumor cells, and streptavidin 488 conjugate was used for secondary incubation (10 μg/mL, Invitrogen). Data were analyzed using FlowJo v10.8 (LLC) and histograms are shown as normalized to mode.

### 4.5. Antibody Dependent Cellular Cytotoxicity Assay (ADCC)

Tumor cells were incubated for 1 h and 30 min with 100 μCi radioactive chromium 51 (^51^Cr, PerkinElmer, Waltham, MA, USA), washed twice with PBS and resuspended in HEPES+ medium [41]. In case of sialidase treatment (Neuraminidase from Vibrio cholerae, Type II, enzyme catalytic activity was evaluated and used at 1 nmol/min, Sigma Aldrich), tumor cells were incubated for 30 min after incubation with ^51^Cr. Neutrophils were stimulated overnight with G-CSF/IFNγ or for 30 min with GM-CSF. In the former condition, cell concentration was corrected for the percentage of apoptotic neutrophils by Annexin V staining (BD Biosciences) and cells were resuspended in HEPES+ medium. Neutrophils and tumor cells were incubated for 4 h at 37 °C and 5% CO_2_ in a ratio of 50:1, respectively, after which incubation was stopped and supernatant was harvested. ^51^Cr release in the supernatant was measured in a gamma counter (Wallac, Gungahlin, ACT, Australia) or a microbeta2 reader (PerkinElmer). Cytotoxicity was evaluated as [(experimental release–spontaneous release)/(maximum release–spontaneous release)] × 100%.

### 4.6. Trogocytosis Assay Using Flow Cytometry

We used flow cytometry to measure the capacity of neutrophils to trogocytose tumor cells [25]. Then, 5 × 10^6^ per mL neutrophils primed with G-CSF/IFNγ for 4 h were labelled with Cell-Trace Calcein Red-Orange AM fluorescent dye (0.4 μg/mL, Thermo Fisher Scientific) and 1 × 10^6^ per mL tumor cells were labelled with lipophilic membrane dye DiD (5 μM, Invitrogen) for 30 min at 37 °C. Cells were co-incubated in a 1:5 target-effector ratio for 1 h and 30 min at 37 °C and 5% CO_2_. Trogocytosis was stopped using fixation buffer (0.5% (*w*/*v*) paraformaldehyde (PFA), 1% (*w*/*v*) bovine serum albumin (BSA, Sigma Aldrich) and 20 mM NaF (Merck) and measured by flow cytometry (LSR II, BD Biosciences). Gating strategy was performed as follows: Gating on single cells followed by gating on Calcein Red-Orange-positive cells, followed by gating on DiD-positive events. Data were analyzed using FlowJo v10.8 (LLC).

### 4.7. Adhesion Assay

Neutrophil adhesion was assessed as previously described [54]. Neutrophils (5 × 10^6^ /mL) were incubated with calcein AM (1 μΜ final concentration, Molecular Probes) for 30 min at 37 °C. Neutrophils were washed twice with PBS and reconstituted in 2 × 10^6^ /mL in HEPES+ medium. Where indicated, neutrophils were pre-incubated with antibodies against FcγRIIa, SIGLEC-5/14 or CD18 for 15 min at room temperature. Then, 80 μL of labelled neutrophils were added to an uncoated 96-well MaxiSorp plate (Nunc) and remained unstimulated or were incubated with phorbol myristate acetate (PMA; 100 ng/mL, Sigma Aldrich) for 30 min at 37 °C and 5% CO_2_ (final volume 100 μL). Adhesion was determined in a Genios plate reader after lysis of adherent cells in 0.5% (*w*/*v*) Triton X-100 (Sigma Aldrich) for 5 min at room temperature at an excitation wavelength of 485 nm and an emission wavelength of 535 nm. Adhesion was finally calculated as (lysis of the experimental condition/total cell lysis) × 100%.

### 4.8. Conjugate Formation Assay Using Image Stream

Neutrophils (5 × 10^6^ /mL) were primed for 4 h with G-CSF/IFNγ, as mentioned previously [25]. Neutrophils and A431 cells were labelled with Cell Trace calcein violet AM fluorescent dye (0.7 μg/mL, Invitrogen) or CMTPX/cell tracker red fluorescent dye (4 μg/mL, Invitrogen), for 30 min at 37 °C and 5% CO_2_, respectively. Effector and tumor cells were co-incubated for 45 min at 37 °C and 5% CO_2_ in a ratio of 5:1 and the assay was stopped using 3.7% PFA for 10 min at 4 °C. Finally, cells were resuspended in 30 μL PBS and conjugate formation was determined using an ImageStreamX flow cytometer (Amnis Corporation, Seattle, WA, USA), acquiring 10,000 images per sample. Gating strategy and data analysis were performed using IDEAS data analysis software (Version 6.3) (Amnis, EMD Millipore, Seattle, WA, USA), as previously described [20].

### 4.9. Flow Cytometry for Detection of CD11b-CD18 Activity

CD11b-CD18 affinity conformation was determined using flow cytometry. Neutrophils were incubated with A431 cells (where indicated A431 cells were opsonized with 10 μg/mL cetuximab) in a ratio of 5:1 for 1 h at 37 °C and 5% CO_2_. Cells were then maintained on ice and stained for the activated epitope of human CD11b (clone CBRM1/5; 20 μg/mL, Thermo Fisher Scientific) and total CD18 (clone MEM48; 10 μg/mL, Diaclone), in one incubation step. CD11b CBRM1/5 antibody was conjugated with fluorescent label 633 using the Lightning-Link^®^ Rapid Atto633 Antibody Labeling Kit (Novus Biologicals, Cambridge, UK). CD11b-CD18 activation was calculated as the ratio of geometric mean fluorescent intensity of CD11b active epitope to the geometric mean fluorescent intensity of CD18. Data were analyzed using FlowJo v10.8 (LLC).

### 4.10. Neutrophil Binding Avidity Assay

In the presence or absence of cetuximab (10 μg/mL) A431 cells were seeded on poly-L-lysine-coated temperature-controlled microfluidic chips for 2 h prior to testing on the z-Movi^®^ Cell Avidity Analyzer (LUMICKS CA B.V., Amsterdam, The Netherlands). Neutrophils were pre-stimulated with G-CSF/IFNγ for 1 h prior use and then labelled with Cell Trace far-red fluorescent dye (0.5 μg/mL, Thermo Fisher Scientific) for 20 min at 37 °C. Neutrophils were pre-incubated with antibodies against CD18 (clone IB4; 20 μg/mL, ATCC), SIGLEC-5/14 (10 μg/mL, R&D systems), or both, for 15 min at room temperature, where indicated. After co-incubation of neutrophils with the seeded A431 cells for 5 min, acoustic force for cell detachment was performed (1000 pN relative force over 150 s), using the z-Movi Cell Avidity Analyzer (LUMICKS CA B.V.). The experiments were performed in triplicates, at 37 °C. Cell avidity was analyzed using Oceon software (https://lumicks.com/knowledge/the-z-movi-workflow/; accessed on 2 August 2022) (LUMICKS CA B.V.).

### 4.11. Human Tumor RNA-Sequencing Analysis

RNA expression levels of SIGLEC-5 or SIGLEC-14 immune receptors and CMAS, SLC35A1, ST3GAL1-6, ST6GAL1-2, ST6GALNAC1-2, 5-6, and ST8SIA1-2, 4-6 enzymes in tumor tissues from breast, ovarian, or pancreatic cancer as well as melanoma and colon carcinoma were assessed using TCGA and TARGET. Expression levels were matched with healthy tissues collected from Genotype-Tissue Expression Project (GTEX). Data were downloaded as log2 normalized to healthy tissue from UCS Xena (https://xenabrowser.net/; accessed on 10 October 2020) [55]. Patients for whom expression of the abovementioned enzymes was not evaluated were omitted. Data were analyzed using Graph Pad Prism 8.1.

### 4.12. Statistics and Data Analysis

Statistical analysis was performed using Graph Pad Prism 9.1.1. Significance among two groups was evaluated using paired *t*-test. Significance among more than two groups was evaluated using one-way ANOVA with Sidak or Tukey correction test. Every figure caption includes the significance test performed. Asterisks indicate statistical significance, where *p* values less than 0.0001 are indicated by ****, *p* values < 0.01: **, *p* values < 0.05: *, and *p* values > 0.05 (not significant, ns).

## Data Availability

All data generated and analysed during this study are available from the corresponding author upon reasonable request.

## Supplementary Materials

The following supporting information can be downloaded at: https://www.mdpi.com/article/10.3390/ijms242417141/s1.
