# Supplementary material for: SIGLEC-5/14 Inhibits CD11b/CD18 Integrin Activation and Neutrophil-Mediated Tumor Cell Cytotoxicity
Source: Int J Mol Sci. 2023 Dec 5;24(24):17141. doi: 10.3390/ijms242417141 (PMC10742634; doi:10.3390/ijms242417141)
Supplement: Supplementary file 1 [file ijms-24-17141-s001.zip › ijms-2685183-supplementary.pdf]

Supplemental Figure S1

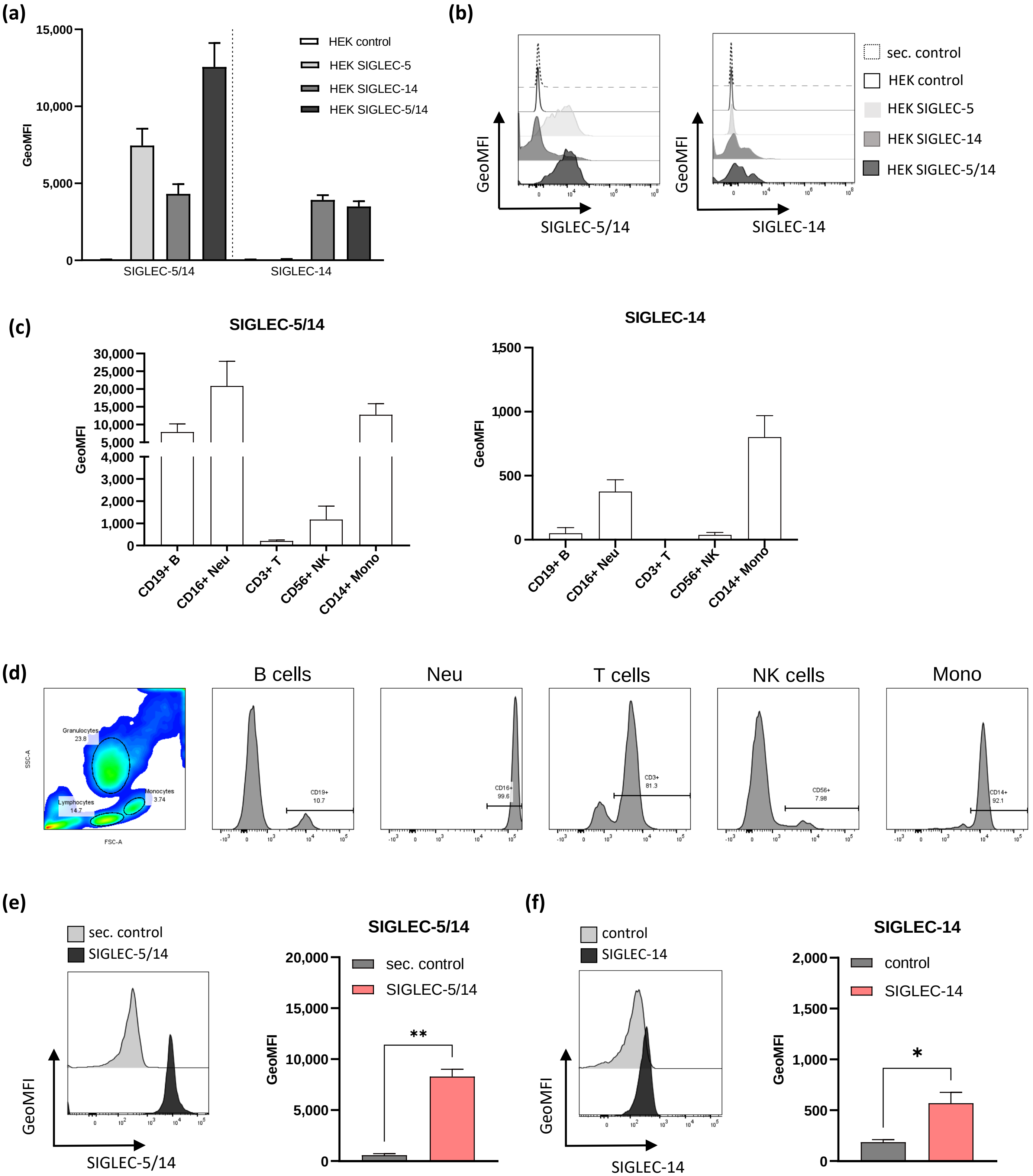

Supplemental Figure S1. SIGLEC-5/14 antibody characterization and protein expression by primary cells. **(a):** Geometric mean fluorescent intensity (GeoMFI) of the expression of human SIGLEC-5/14 and SIGLEC-14 after transfection of *SIGLEC-5*, *SIGLEC-14* or both genes in HEK293T cells *(3 independent transfection experiments)*. GeoMFI is corrected to secondary control. **(b):** Histograms from one representative example depict the expression of SIGLEC-5/14 or SIGLEC-14. **(c):** Graphs represent the geometric mean fluorescent intensity (GeoMFI) for the expression of SIGLEC-5/14 (left panel) or SIGLEC-14 (right panel) for the whole blood-derived cells *(3 donors from 3 independent experiments)*. GeoMFI is corrected to secondary control or isotype control. **(d):** Forward-side scatter dot plot from one representative donor depicts the initial gating for granulocytes, lymphocytes, or monocytes. Histograms from one representative example depict the gating strategy for identification of whole blood- derived CD19-positive B cells, CD16-positive neutrophils, CD3-positive T cells, CD56-positive NK cells or CD14-positive monocytes. **(e) - (f):** GeoMFI of the expression of SIGLEC receptors by isolated neutrophils in steady state *(4 donors from 2 independent experiments)*. Representative histograms indicate SIGLEC expression. Bars show mean± SEM. Statistics: unpaired t-test; *, $p<0.05$; **, $p<0.01$. Scr, Scrambled.

Supplemental Figure S2

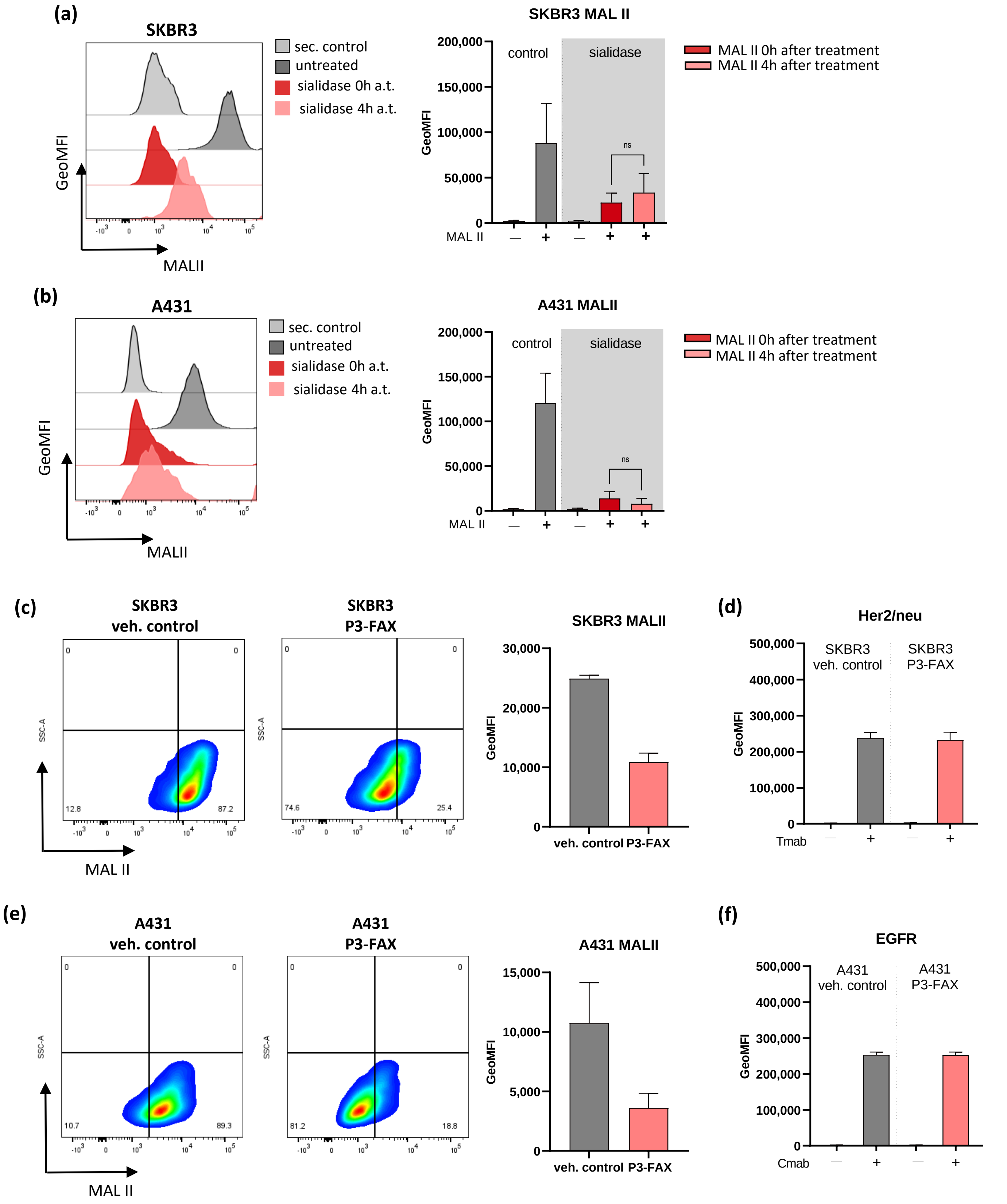

Supplemental Figure S2

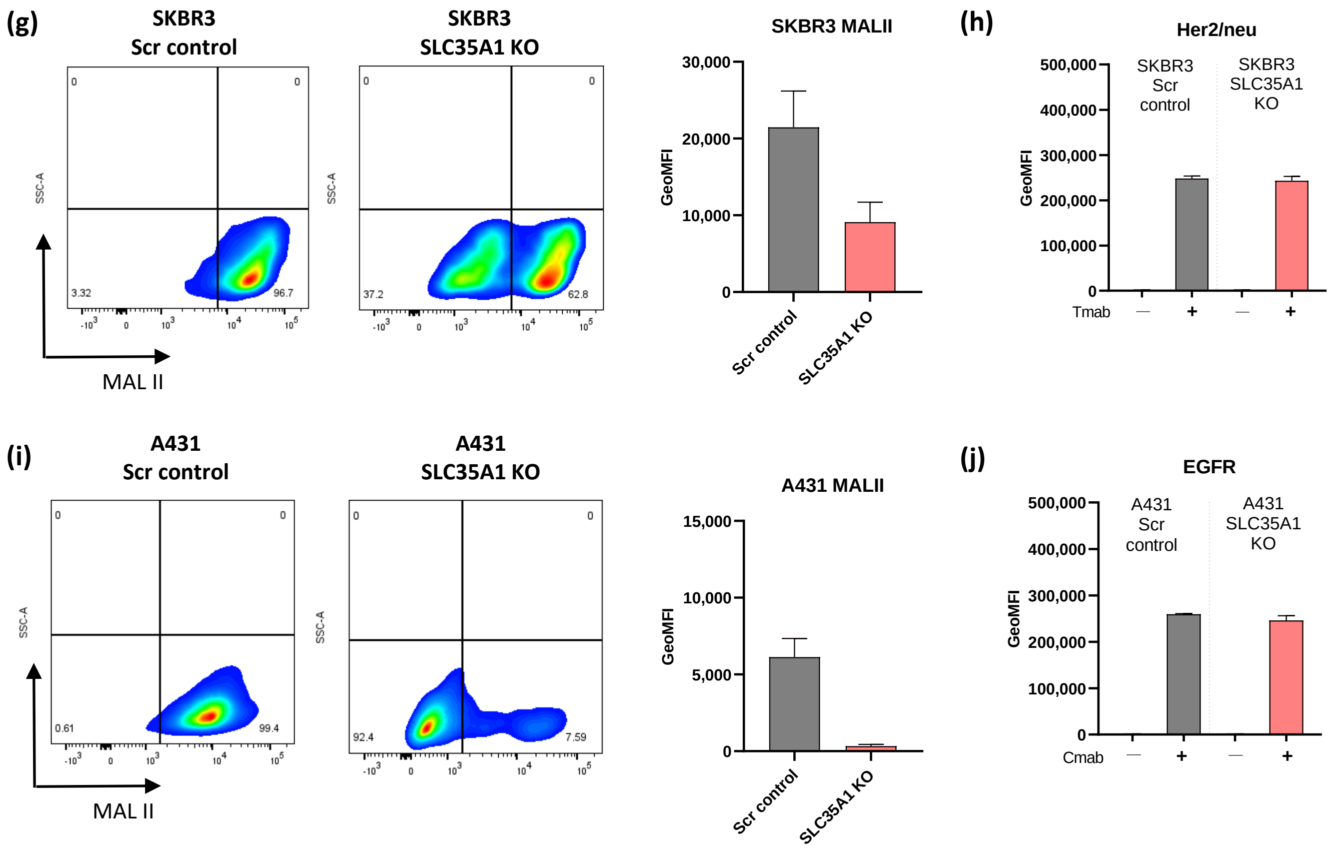

Supplemental Figure S2. Sialic acid expression on tumor cells

(a) –(f) Binding of maackia amurensis lectin II (MAL II) to SKBR3 (a, c, g) or A431 cells (b, d, i). **(a)-(b):** Geometric mean fluorescent intensity (GeoMFI) of MAL II binding to SKBR3 (a) or A431 (b) cells treated with sialidase. MAL II lectin binding was tested immediately (red bar) or 4 hours after treatment (pink bar) *(3 independent experiments)*. Histograms show one representative experiment and are depicted as normalized to mode. **(c) – (j)** Characterization of vehicle control or P3-FAX- treated cells (**c-f**) or SLC35A1KO cells (**g-j**). Dot plots depict the MAL II lectin binding to control or modified cells. Panels depict the GeoMFI for the binding of MAL II lectin to control untreated (grey) or modified (pink) tumor cells (**c, e, g, i**). GeoMFI is corrected to secondary control staining. **(d), (f), (h), (j)** Panels depict the expression of Her2/neu or EGFR tumor antigen by SKBR3 or A431 cells as GeoMFI, respectively, before and after treatment with P3-FAX or SLC35A1 deletion *(d, f: 2 independent experiments; h, j: 3 independent experiments)*. Bars show mean± SEM. Statistics: unpaired t-test; ns, not significant. Tmab, trastuzumab; Cmab, cetuximab.

Supplemental Figure S3

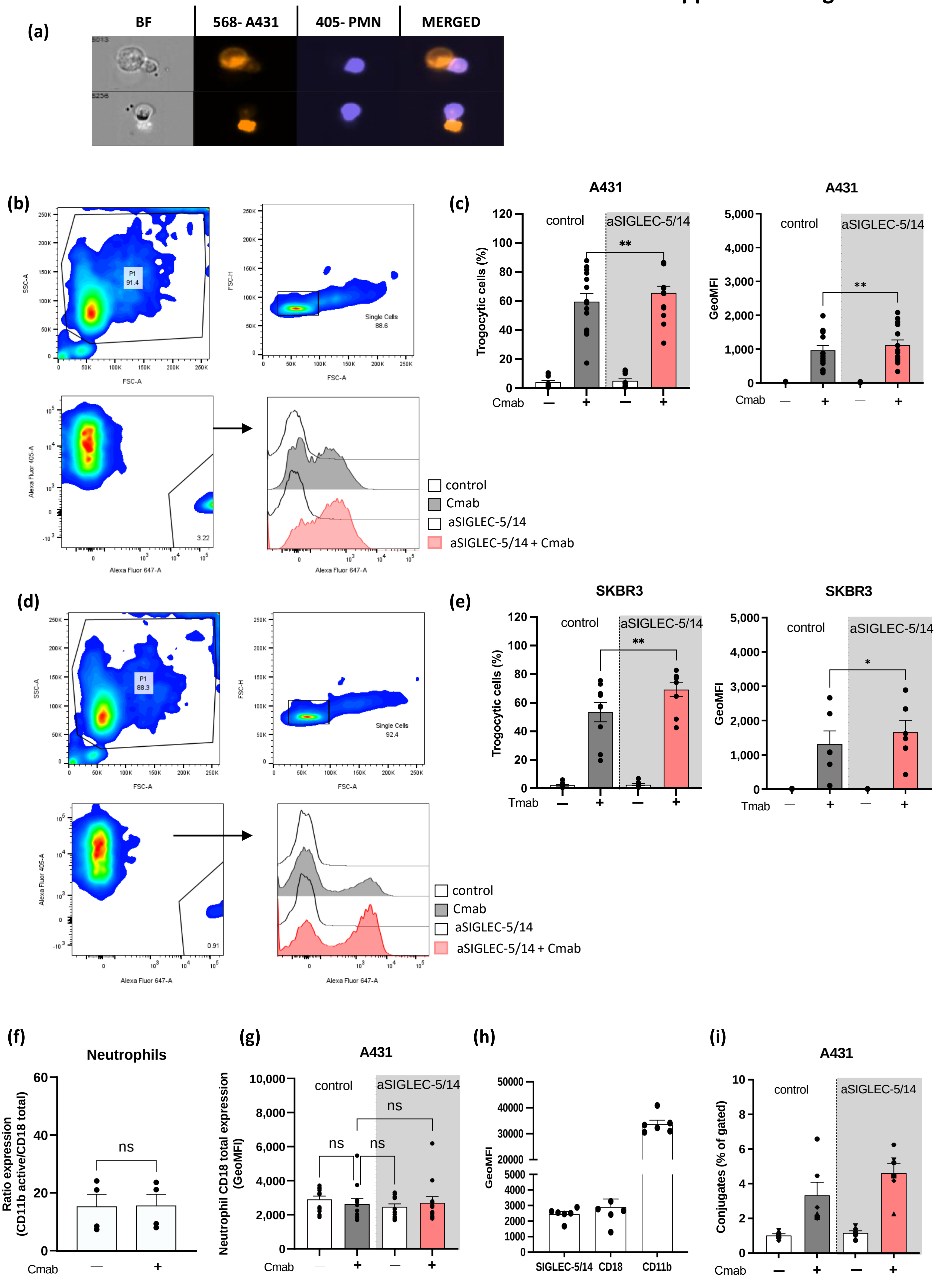

Supplemental Figure S3. SIGLEC-5/14 inhibition during conjugate formation or trogocytosis

(a) Representative example of conjugate formation of A431 cells opsonized with cetuximab and neutrophils pre-incubated with SIGLEC-5/14 blocking antibody. **(b) – (c)** Trogocytosis of A431 cells in the presence or absence of cetuximab, where neutrophils were pre-incubated with SIGLEC-5/14 blocking antibody *(9 donors from 4 independent experiments)*. Results indicate the percentage of neutrophils positive for trogocytosis (b) or the geometric mean fluorescent intensity (GeoMFI) of trogocytic cells (c). **(d) – (e)** Neutrophil trogocytosis against SKBR3 cells in the presence or absence of opsonizing antibody trastuzumab and SIGLEC-5/14 antibody. Results show the percentage of neutrophils positive for trogocytosis (d) or geometric mean fluorescent intensity (GeoMFI) of the trogocytic cells (e) *(9 donors from 4 independent experiments)*. **(f)** Graph depicts the ratio of the GeoMFI expression for the activated epitope of CD11b (CBRM1/5) to the total CD18 expression on neutrophils in the presence or absence of cetuximab *(4 donors from 2 independent experiments).* **(g)** Total CD18 expression on neutrophils in the presence or absence of SIGLEC-5/14 blocking antibody and cetuximab-opsonized A431 cells *(12 donors from 6 independent experiments)*. **(h)** GeoMFI expression of CD11b, CD18 or SIGLEC-5/14 for the neutrophils from 6 donors which were used for the avidity assay. **(i)** Conjugate formation of 6 donors used for avidity assay. Statistics: **(b) – (e):** paired t-test; **(g)** one way ANOVA with Sidak correction; ns, nonsignificant; *, $p<0.05$; **, $p<0.01$. Tmab, trastuzumab; Cmab, cetuximab; BF, bright-field.
